# Asp-ase Activity of the Opossum Granzyme B Supports the Role of Granzyme B as Part of Anti-Viral Immunity Already during Early Mammalian Evolution

**DOI:** 10.1371/journal.pone.0154886

**Published:** 2016-05-06

**Authors:** Zhirong Fu, Michael Thorpe, Srinivas Akula, Lars Hellman

**Affiliations:** Department of Cell and Molecular Biology, Uppsala University, Uppsala, The Biomedical Center, Box 596, SE-751 24 Uppsala, Sweden; University of Gdansk, POLAND

## Abstract

Granzyme B is one of the key effector molecules in our defense against viruses and intracellular bacteria. This serine protease together with the pore forming protein perforin, induces caspase or Bid-dependent apoptosis in target cells. Here we present the first characterization of a granzyme B homolog, the grathepsodenase, in a non-placental mammal, the American opossum (*Monodelphis domestica*). The recombinant enzyme was produced in a human cell line and used to study its primary and extended cleavage specificity using a panel of chromogenic substrates and recombinant protein substrates. The opossum granzyme B was found to have a specificity similar to human granzyme B, although slightly less restrictive in its extended specificity. The identification of a granzyme B homolog with asp-ase (cleaving after aspartic acid) specificity in a non-placental mammal provides strong indications that caspase or Bid-dependent apoptosis by a serine protease with a conserved primary specificity has been part of anti-viral immunity since early mammalian evolution. This finding also indicates that an asp-ase together with a chymase were the first two serine protease genes to appear in the mammalian chymase locus.

## Introduction

Secretory granules filled with inflammatory mediators are found in several of the major hematopoietic cell types. In mast cells, NK cells, cytotoxic T cells and neutrophils, serine proteases make up the majority of the protein content of these granules [1]. Mast cells contain chymases and tryptases with chymotrypsin- and trypsin-related primary cleavage specificities [1–3]. Similarly, NK cells and cytotoxic T cells contain a number of granzymes (Gzms) with varying specificities. Human NK cells and cytotoxic T cells express five different Gzms; -A, -K, -M, -H and–B [1]. Granzymes A and K are tryptases, GzmM a met-ase, GzmH a chymase and GzmB an asp-ase, the later cleaving after the negatively charged amino acids, aspartic and glutamic acid. Granzymes have a number of different functions, including induction of apoptosis, triggering of cytokine production and anchoring-dependent cell death termed anoikis [1]. The most prominent role in immunity is likely to be the induction of apoptosis in cells infected with viruses and intracellular bacteria as well as transformed cancerous cells. Both caspase/Bid-dependent and -independent activation of apoptosis has been described for these enzymes. Granzyme B is the most well documented example of the first type, the caspase or Bid-dependent mechanism [4]. Granzyme B is also the only member of the hematopoietic serine proteases, which cleaves after negatively charged amino acids. This enzyme is primarily expressed by cytotoxic T cells, although it is also expressed in mast cells and possibly other cells under certain conditions. However, mast cells do not express perforin, which makes the function of GzmB in this cell type more elusive.

The aim of this study was to determine the appearance of the asp-ase specificity during vertebrate evolution. This may shed light on the importance of caspase/Bid-dependent mechanisms contra non-caspase/Bid dependent mechanisms for our defense against intracellular parasites and tumors. As a first step in this process we present the analysis of the first non-placental mammalian enzyme, an enzyme from the marsupial the American opossum (*Monodelphis domestica*). The enzyme was produced as a recombinant, inactive enzyme in a human cell line (HEK 293 EBNA) and purified from the conditioned media. After activation by enterokinase (EK) cleavage, this enzyme was analyzed for its primary and extended specificity using a panel of chromogenic and recombinant substrates.

**Fig 1:**
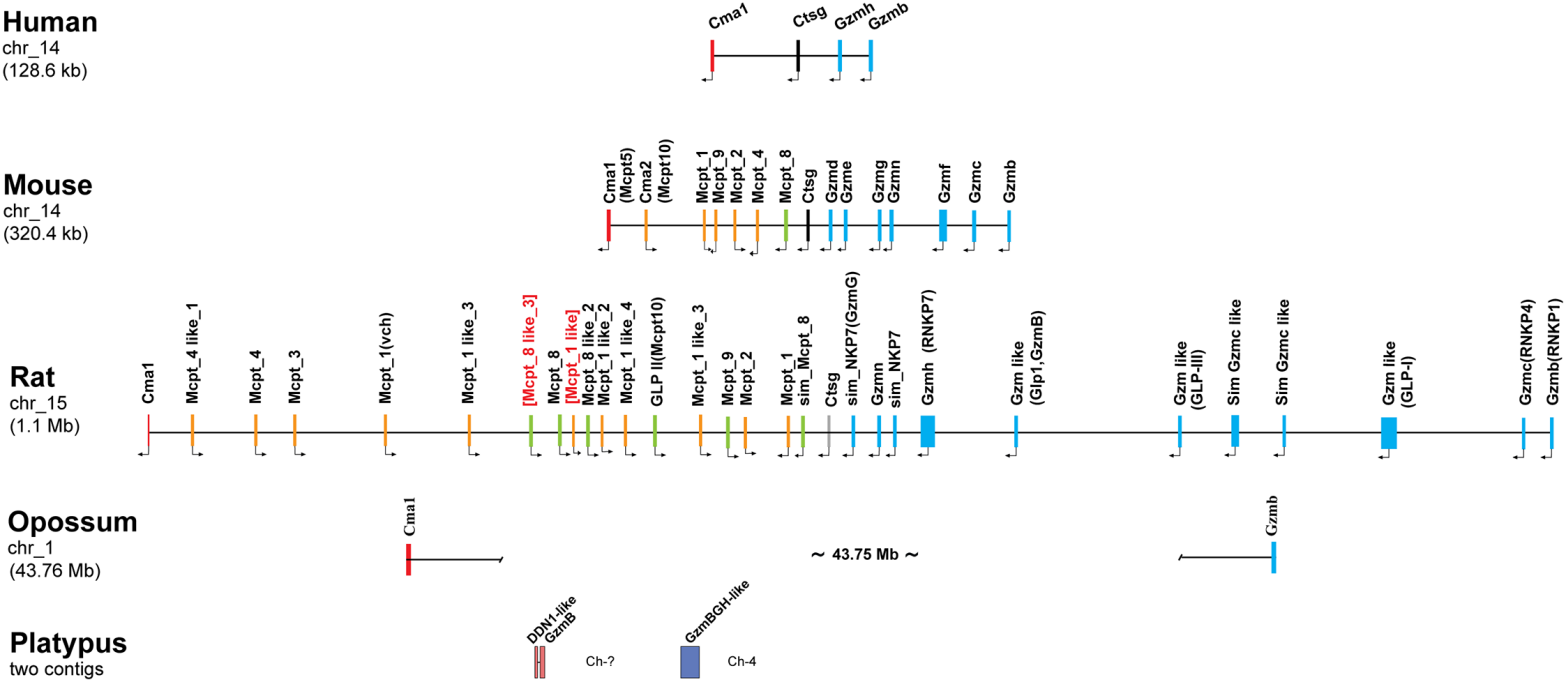
The chymase locus. The chymase loci of selected species are shown to scale in order to visualize the large differences in gene number and size between various mammals from humans to marsupials and monotremes.

Granzyme B originates from the chymase locus. In humans this locus contains four serine protease genes: the mast cell chymase, neutrophil cathepsin G and two T cell expressed Gzms; H and B. Interestingly in rodents, there has been a massive expansion of this locus resulting in 15 functional serine protease genes in mice and 28 in rats [5, 6] (Fig 1). However, in the opossum this chromosomal region has undergone an inversion, which may explain why the locus has not expanded to the same extent as most placental mammals [6, 7] (Fig 1). The opossum has only two chymase locus genes and we have previously shown that one of them is a mast cell chymase homolog [8]. This enzyme has chymotryptic activity with a slightly different primary specificity compared to the human and rodent orthologs [8]. The opossum chymase prefers tryptophan in the P1 (cleavage) position in contrast to the human chymase and the mouse counterpart mMCP-4, which both prefer tyrosine and phenylalanine, respectively [8, 9]. The second enzyme of the chymase locus in the opossum has not been analyzed for its primary or extended specificity, and this was the focus of this study. Interestingly, in all placental mammals studied the chymase locus is bordered at one end by the mast cell chymase and at the other end by GzmB, indicating these two enzymes were the first to appear in this locus.

**Fig 2:**
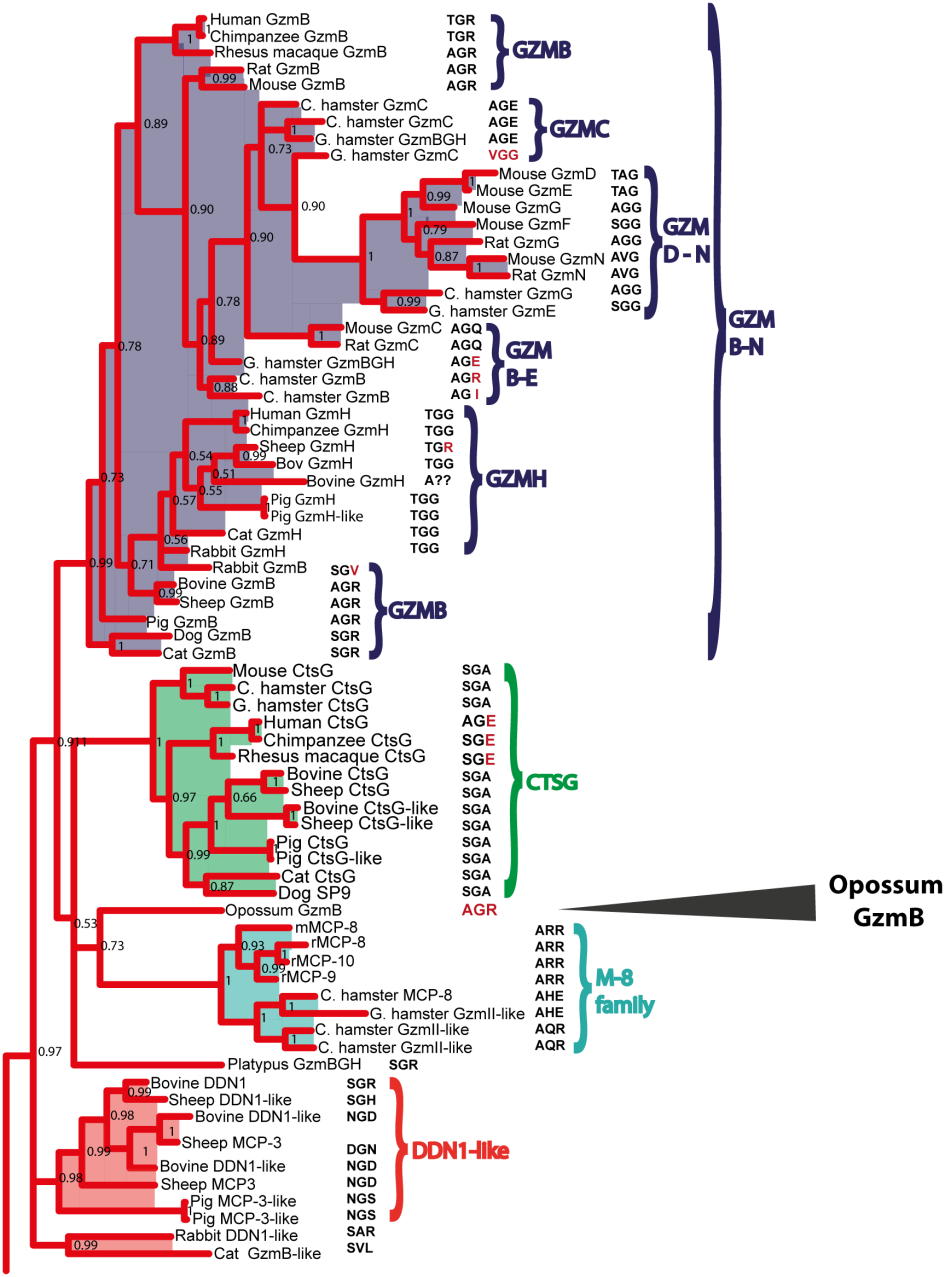
A phylogenetic tree indicating the position of opossum GzmB among related hematopoietic proteases. A modified section of a larger phylogenetic analysis of 386 different serine proteases presented in a recent article on the evolution of hematopoietic serine proteases [19]. It is unclear whether the position of opossum GzmB based on sequence similarity is more closely related functionally to cathepsin G, the mMCP-8 family of serine proteases or the granzymes B, C, D, E, F, G, N or H. The triplets of the catalytic pocket corresponding to the amino acid positions 189, 216 and 226 (according to chymotrypsinogen numbering) is depicted after each enzyme in the tree. Opossum GzmB has the same triplet as mouse and rat GzmB, which are different triplets in comparison to the majority of cathepsin Gs and the mMCP-8 family members, indicating a close functional relation to GzmBs.

In a phylogenetic analysis, the opossum GzmB-like enzyme ended up outside of the group of enzymes formed by cathepsin G, the mMCP-8 related enzymes and the granzymes in placental mammals (Fig 2). This has made its origin and specificity difficult to assign only based on phylogenetics, and also why the opossum enzyme has been named the grathepsodenase [7]. A more detailed analysis of its extended specificity was therefore needed to assign its function in marsupial immunity as well as in the appearance of the different specificities of the enzymes found among the enzymes of the mammalian chymase locus. By using a panel of chromogenic and recombinant substrates, we showed that this enzyme is an asp-ase and therefore most likely a GzmB homolog, albeit with a slightly broader specificity compared to its human counterpart. This also supports the conclusion that the mast cell chymase and GzmB were the first two enzymes to appear in this locus. In order not to cause confusion, the grathepsodenase will be referred to as GzmB from here on.

## Materials and Methods

### Production, purification and activation of recombinant human and opossum GzmB

The opossum and human GzmB sequences (GenBank accession code: AY942183) were designed and ordered from GenScript (Piscataway, NJ, USA). The sequences were subsequently transferred to a pCEP-Pu2 vector used for expression in mammalian cells [10]. The enzymes were produced as inactive recombinant proteins, with an N-terminal His_6_-tag followed by an enterokinase (EK) site.

HEK 293 cells were grown to 70% confluency in a 25 cm^3^ tissue culture flask (BD VWR) with Dulbecco’s Modified Eagles Medium (DMEM) (GlutaMAX, Invitrogen) supplemented with 5% fetal bovine serum (FBS) and 50 μg/ml gentamicin. Following DNA (25 μg of opossum GzmB or human GzmB in pCEP-Pu2) transfection with lipofectamine (Invitrogen, Karlsbad, CA, USA), puromycin was added to the DMEM (0.5 μg/ml) to select for cells, which had taken up the DNA. Heparin was also added to the medium (5 μg/ml) to enhance yield, as these positively charged proteases tend to bind cell surfaces and plastic, where heparin can reduce the loss of the protein. Cells were expanded and conditioned media collected.

To purify the recombinant enzyme, 750 ml conditioned media was filtered (Munktell 00H 150 mm, Falun, Sweden) and 500 μl nickel nitrilotriacetic acid (Ni-NTA) beads were added. The media with Ni-NTA beads were rotated for 45 mins at 4°C. Subsequently, the Ni-NTA beads were collected by centrifugation and transferred to a column containing a glass filter (Sartorius, Goettingen, Germany). After washing with PBS tween 0.05% + 10 mM imidazole + 1 M NaCl, the recombinant protein was eluted in PBS tween 0.05% + 100 mM imidazole fractions. The first fraction volume was half the Ni-NTA bead width (200 μl) and further fractions eluted with a full bead width (400 μl). Individual fractions were run on SDS-PAGE gel, their concentrations estimated from a bovine serum albumin standard and the most concentrated were pooled and kept at 4°C.

**Fig 3:**
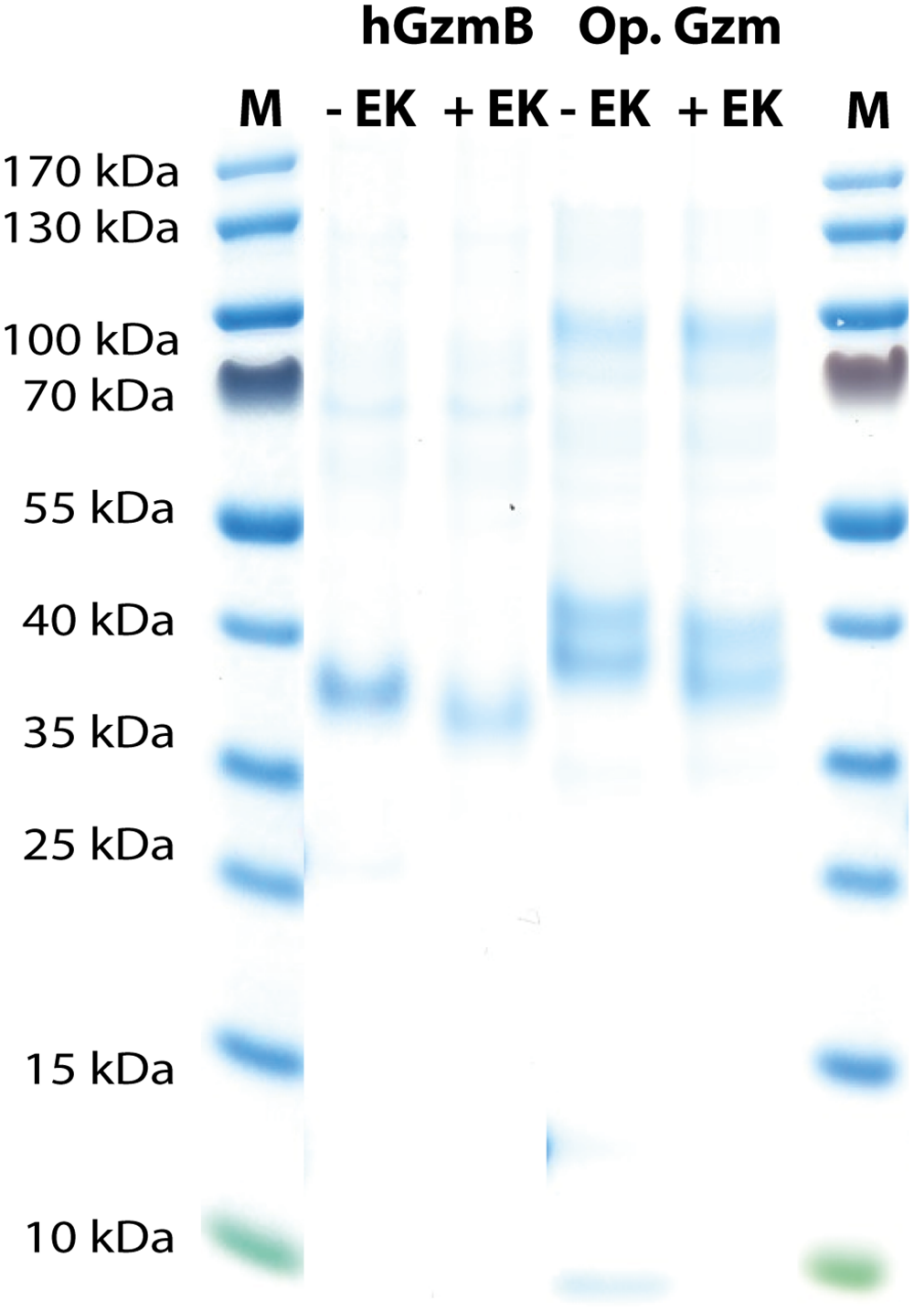
Purification and activation of recombinant opossum grathepsodenase (GzmB) and human GzmB. The two proteases were expressed in the human HEK 293 EBNA cell line. The proenzymes (-EK) were first purified from the conditioned media of transfected cells using Ni-NTA beads and then activated by removal of the His_6_-tag by enterokinase digestion (+EK). The purified enzyme both before and after enterokinase cleavage was analyzed on SDS-PAGE and visualized with Coomassie Brilliant Blue staining.

The recombinant opossum GzmB, and human GzmB initial concentrations were determined by SDS-PAGE and the level of EK (Roche, Mannheim, Germany) adjusted for activation of the enzyme. A relative concentration was activated depending when it was needed, for example 70 μl of the eluted recombinant enzyme was digested with 1 μl EK for 3 hrs at 37°C (Fig 3). The activated fractions were stored at 4°C until use. The identity of the opossum enzyme was confirmed by mass spectrometry after trypsin digestion performed at the SciLife Lab Mass Spectrometry Facility in Uppsala, Sweden (S1 Fig).

### Analysis of the primary specificity by the use of chromogenic substrates

**Fig 4:**
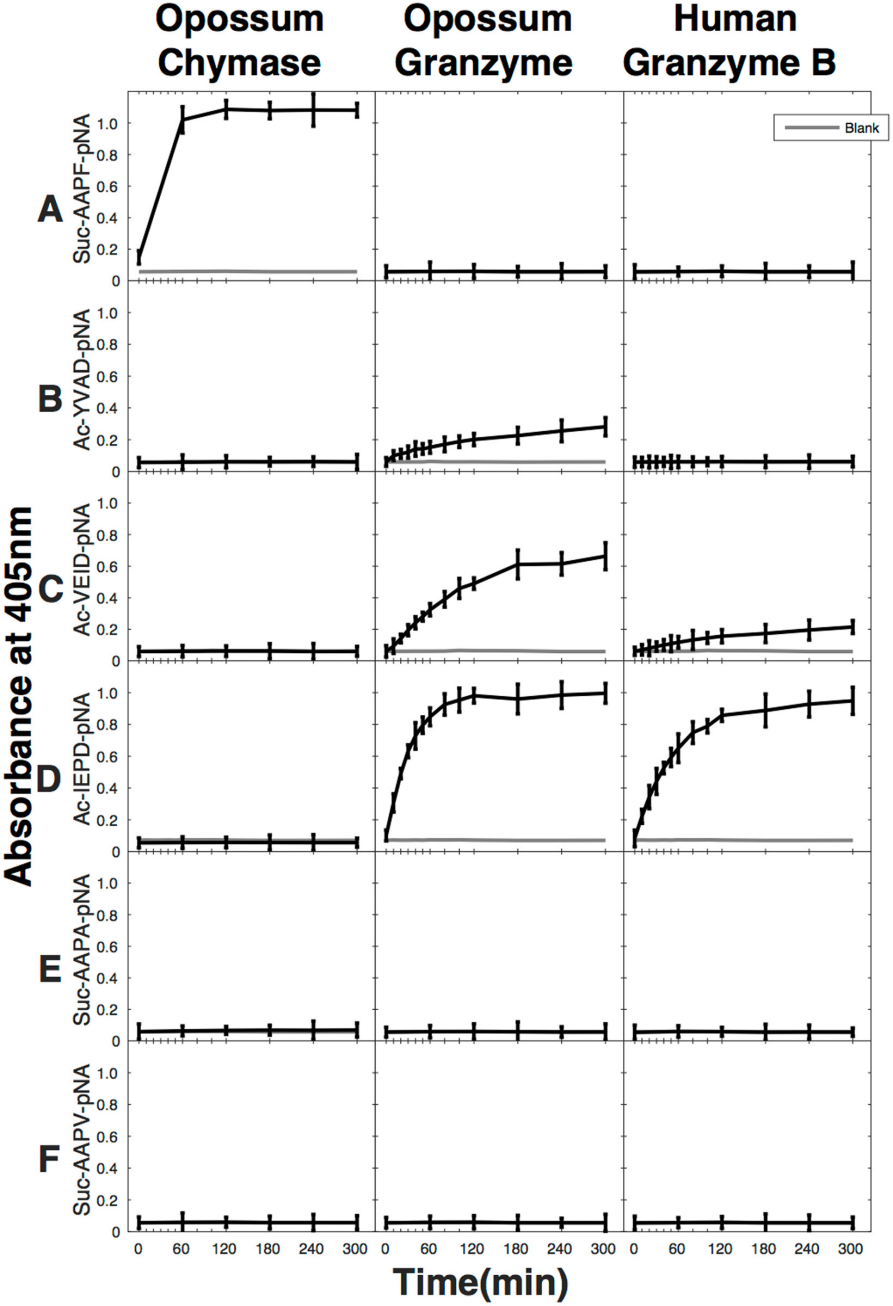
Chromogenic substrate assay of three recombinant enzymes: opossum chymase, opossum grathepsodenase (GzmB) and human GzmB. The cleavage of the substrates in 200 μl reaction volumes were measured in a spectrophotometer during a 5 hour incubation course. The optical density (OD) values, at 405 nM, for each hour were plotted.

A panel of different chromogenic substrates were used to study the primary specificities of the three enzymes: the opossum chymase, the opossum GzmB and human GzmB. A chymase substrate (Suc-AAPF-pNA), three asp-ase substrates (Ac-IEPD-pNA) (Ac-VEID-pNA) and (Ac-YVAD-pNA) and two elastase substrates (Suc-AAPV-pNA and Suc-AAPA-pNA) were used (Fig 4). Additionally five substrates were also tested: two tryptase (Boc-VLGR-pNA and Z-GPR-pNA) one additional elastase (Suc-AAPI-pNA), one with a cleavable leucine (Suc-AAPL-pNA) and one additional chymase substrate (Suc-LLVY-pNA) (S2 Fig). All substrates were purchased from Bachem AG (Bubendorf, Schweiz). The reactions were run in a total volume of 200 μl in a micro titer plate. The substrate concentration was 0.2 mM and 200 ng of the activated enzymes were used. All reactions were run in a phosphate buffered saline pH 7.5.

### Generation of recombinant substrates for the analysis of the cleavage specificity

**Fig 5:**
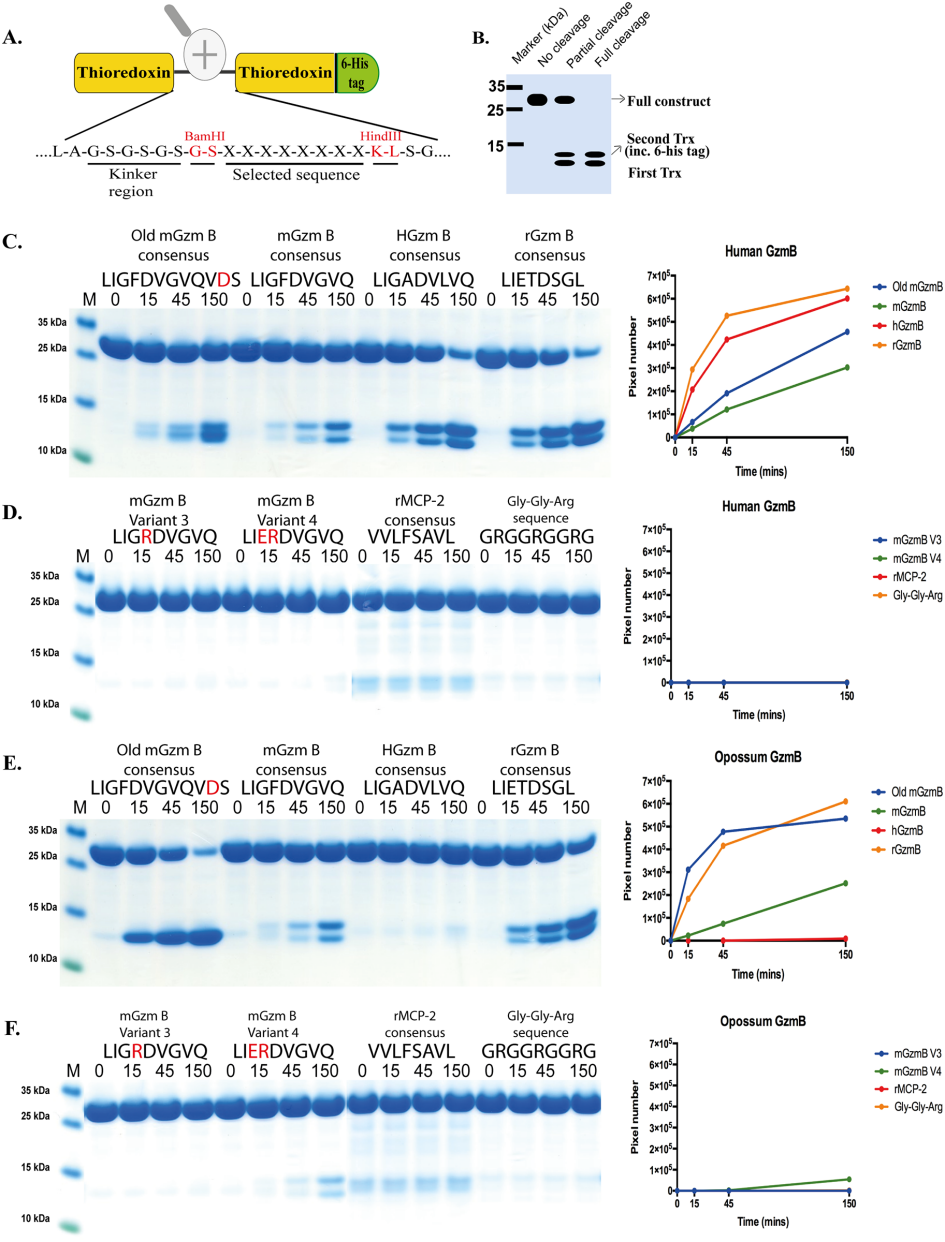
Analysis of a set of recombinant substrates. Panel A shows the overall structure of the recombinant protein substrates used for analysis of the efficiency in cleavage by human and opossum GzmB. In these substrates two thioredoxin molecules were positioned in tandem and the proteins have a His_6_-tag positioned in their C termini. The different cleavable sequences were inserted in the linker region between the two thioredoxin molecules by the use of two unique restriction sites, one Bam HI and one HindIII site, which are indicated in the bottom of panel A. The name and sequence of the different substrates are indicated above the gel images. The time of cleavage (in minutes) is also indicated above the corresponding lanes of the different gels. The uncleaved substrates have a molecular weight of approximately 25 kDa and the cleaved substrates appear as two closely located bands with a size of 12–13 kDa. The residues in the mouse variants that differ from the mouse GzmB consensus are marked in red. In addition in panels C and E the mouse GzmB consensus with the extra Asp of the SalI site has been included as a reference. Here the extra Asp is also marked in red. The product bands were scanned and plotted in diagrams that are shown to the right of each gel panel.

**Fig 6:**
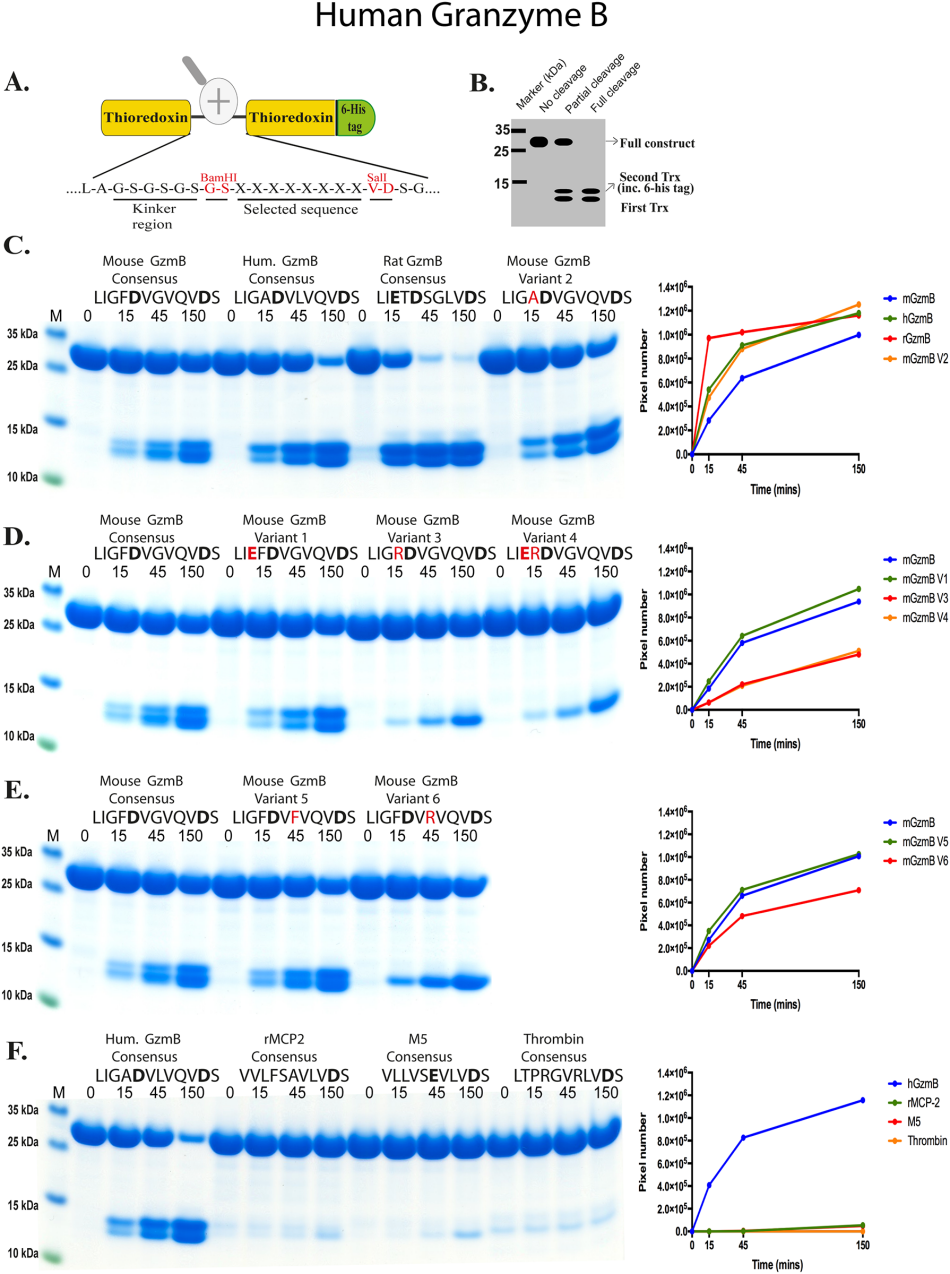
Analysis of the cleavage specificity of human GzmB by the use of recombinant protein substrates containing an extra Asp residue encoded in the SalI site of the vector. Panel A shows the overall structure of the recombinant protein substrates used for analysis of the efficiency in cleavage by human GzmB. The different cleavable sequences were inserted in the linker region between the two thioredoxin molecules by the use of two unique restriction sites, one Bam HI and one SalI site, which are indicated in the bottom of panel A. Panel B shows a schematic drawing of the cleavage of a recombinant substrate. The uncleaved substrates have a molecular weight of approximately 25 kDa and the cleaved substrates appear as two closely located bands with a size of 12–13 kDa. Panels C to F show the cleavage of a number of substrates by human GzmB. The name and sequence of the different substrates are indicated above the pictures of the gels. The negatively charged amino acids, E or D, are marked in bold. The time of cleavage in minutes is also indicated above the corresponding lanes of the different gels. The residues in the mouse variants that differ from the mouse GzmB consensus are marked in red. The product bands were scanned and plotted in diagrams that are shown to the right of each gel panel.

A new type of substrate was developed to expand on the results obtained from the chromogenic analysis. Two copies of the *E*. *coli* thioredoxin gene were inserted in tandem into the pET21 vector for bacterial expression (Fig 5A). In the C-terminal end a His_6_- tag was inserted for purification on Ni^2+^ IMAC columns. In a linker region between the two thioredoxin molecules, different substrate sequences were inserted by ligating double stranded oligonucleotides into two unique restriction sites, one BamHI and one HindIII site (Fig 5A), or an alternative vector with one BamHI site and one SalI site (Fig 6A). The sequences of the individual clones were verified after cloning by sequencing of both DNA strains. The plasmids were then transformed into the *E*.*coli* Rosetta gami strain for protein expression (Novagen, Merck, Darmstadt, Germany). A 10 ml overnight culture of the bacteria harboring the plasmid was diluted 10 times in LB + Amp and grown at 37°C for 1–2 hours until the OD (600 nm) reached 0.5. IPTG was then added to a final concentration of 1 mM. The culture was then grown at 37°C for an additional 3 hours under vigorous shaking, after which the bacteria were pelleted by centrifugation at 3500 rpm for 12 minutes. The pellet was washed once with 25 ml PBS + 0.05% Tween 20. The pellet was then dissolved in 2 ml PBS and sonicated 6 x 30 seconds to open the cells. The lysate was centrifuged at 13000 rpm for 10 minutes and the supernatant was transferred to a new tube. Five hundred μl of Ni-NTA slurry (Qiagen, Hilden, Germany) was added and the sample slowly rotated for 45 min at 4°C. The sample was transferred to a 2 ml column and the supernatant was allowed to slowly pass through the filter leaving the Ni-NTA beads with the bound protein in the column. The column was washed four times with 1 ml of washing buffer (PBS + 0.05% Tween + 10 mM Imidazole + 1 M NaCl). Elution of the protein was performed by adding 150 μl elution buffer followed by five 300 μl fractions of elution buffer (PBS + 0.05% Tween 20 + 100 mM Imidazole). Each fraction was collected individually. Ten μl from each of the eluted fractions was then mixed with 1 volume of 2 x sample buffer and 1 μl β-mercaptoethanol and heated for 3 min at 80°C. The samples were analyzed on a SDS bis tris 4–12% PAGE gel and the fractions containing the most protein were pooled. The protein concentration of the combined fractions was determined by Bio-Rad DC Protein assay (Bio-Rad Laboratories Hercules, CA USA). Approximately 60 μg of recombinant protein was added to each 120 μl cleavage reaction (in PBS). Twenty μl from this tube was removed before adding the enzyme, the 0 minute time point. The active enzyme was then added and the reaction was kept at room temperature during the entire experiment. Twenty μl samples were removed at the indicated time points (15 min, 45 min and 150 min) and stopped by addition of one volume of 2x sample buffer. One μl β–mercaptoethanol was then added to each sample followed by heating for 3 min at 80°C. Twenty μl from each of these samples was then analyzed on 4–12% pre-cast SDS-PAGE gels (Invitrogen, Carlsbad, CA, USA). The gels were stained overnight in colloidal Coomassie staining solution and de-stained for several hours as previously described [11]. All protein gels were analyzed using the UN-SCAN-IT Gel Analysis Software from Silk Scientific Inc. (Orem, Utah USA).

## Results

### Production, purification and activation of a recombinant human and opossum GzmB

DNA constructs containing the coding regions for the opossum GzmB and human GzmB, both with an N-terminal His_6_-tag followed by an EK site were designed and ordered from Genscript. These fragments were cloned into the mammalian expression vector pCEP-Pu2 for expression in human embryonic kidney cells, HEK 293 EBNA cells [10]. The His_6_-tag facilitates purification on Ni^2+^ chelating immobilized metal ion affinity chromatography columns and cleavage with EK activates the enzyme, whilst simultaneously removing the His_6_-tag. Samples of inactive and activated protease were separated on SDS-PAGE gels in order to ensure successful removal of the His_6_-tag and the EK susceptible cleavage site (Fig 3). The inactive human GzmB migrated as 38 kDa bands and the EK digested enzyme was slightly smaller around 37 kDa (Fig 3). The inactive opossum GzmB was larger (39–42 kDa) and several bands were seen, probably representing differences in the amounts of N-linked carbohydrates (Fig 3). The opossum GzmB recombinant protein was analyzed by mass spectrometry and the range of observed peaks matched the expected sequence, confirming the correct enzyme was produced (S1 Fig).

### Determination of the primary cleavage specificity by chromogenic substrates

A panel of chromogenic substrates was used to characterize the primary specificity of the opossum GzmB. Human GzmB and the opossum mast cell chymase were used for comparison, as these enzymes have been relatively well characterized. Human GzmB provides a reference for an enzyme with a relatively narrow asp-ase activity. It is also of major interest to compare the enzymes concerning their primary and extended specificities as a step in understanding the evolution of the caspase/Bid-dependent activation of apoptosis in target cells. As seen in Fig 4, the opossum mast cell chymase only cleaved the chymase substrate (Suc-AAPF-pNA). Human GzmB only cleaved two of the asp-ase substrates, one efficiently (Ac-IEPD-pNA) and one less efficiently (Ac-VEID-pNA) but not a third asp-ase substrate (Ac-YVAD-pNA) indicating that substrates with two negatively charged amino acids in the P1 and P3 positions are favored over substrates with only one negative charge (Fig 4). In contrast, opossum GzmB cleaved all three asp-ase substrates but not the chymase or two elastase substrates (Suc-AAPV-pNA and Suc-AAPA-pNA) (Fig 4). This indicates a broader specificity against different asp-ase substrates for the opossum GzmB compared to human GzmB. Five additional chromogenic substrates were also tested: two tryptase (Boc-VLGR-pNA and Z-GPR-pNA) one additional elastase (Suc-AAPI-pNA), one with leucine (Suc-AAPL-pNA) and one additional chymase substrate (Suc-LLVY-pNA). All were found to be negative where no cleavage was observed with the opossum GzmB, which shows the relatively strict specificity for negatively charged amino acids in the P1 position for this enzyme (S2 Fig).

### Use of recombinant protein substrates to obtain a more detailed picture of primary and extended specificities

**Fig 7:**
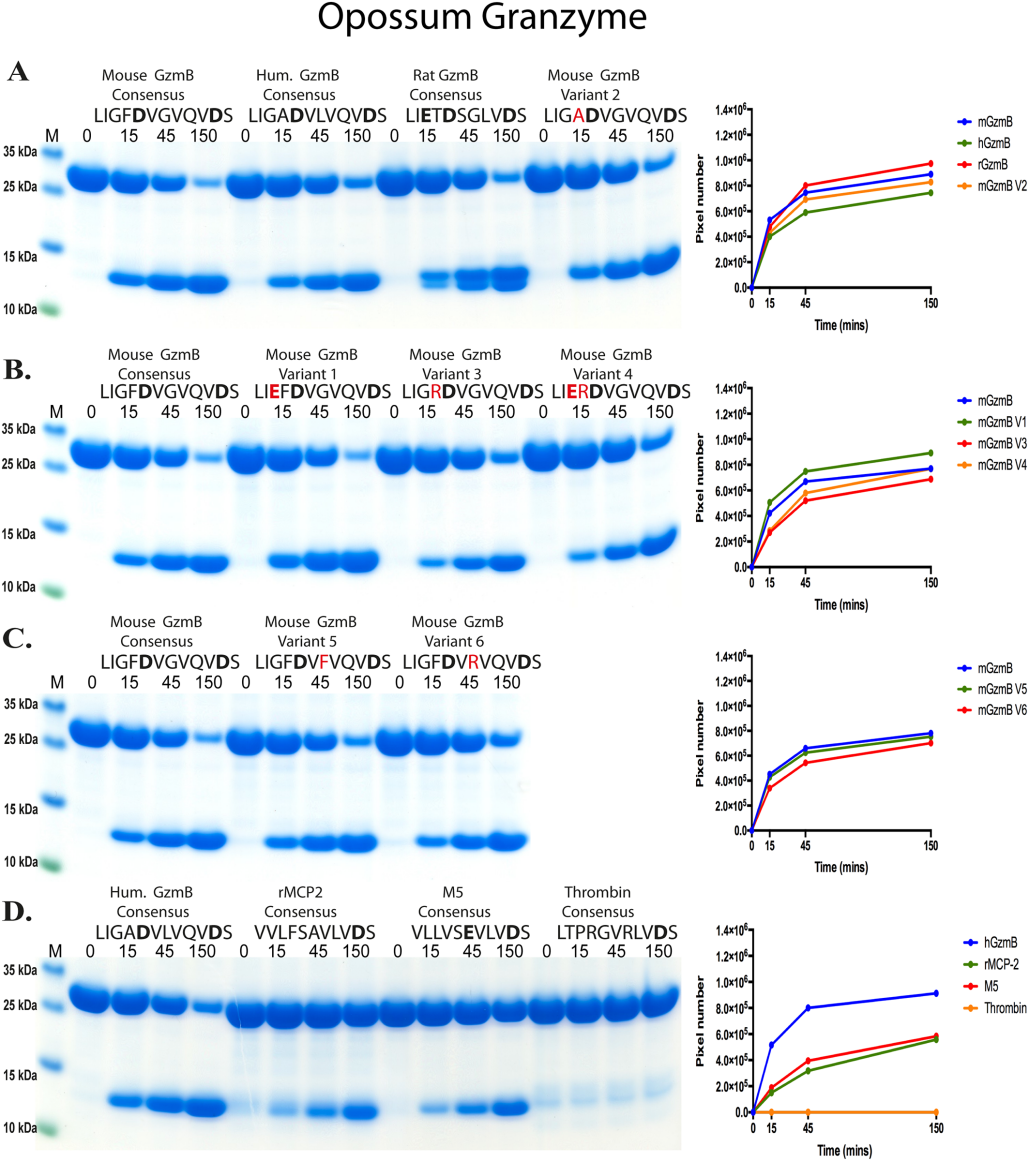
Analysis of the cleavage specificity of the opossum GzmB by the use of recombinant protein substrates cloned with BamHI and SalI and thereby having two or three negatively charged amino acids. Panels A to D show the cleavage of a number of substrates by the opossum GzmB. The name and sequence of the different substrates are indicated above the pictures of the gels. The negatively charged amino acids, E or D, are marked in bold. The time of cleavage in minutes is also indicated above the corresponding lanes of the different gels. The uncleaved substrates have a molecular weight of approximately 25 kDa and the cleaved substrates appear as two closely located bands with a size of 12–13 kDa. The residues in the mouse variants that differ from the mouse GzmB consensus are marked in red. The product bands were scanned and plotted in diagrams that are shown to the right of each gel panel.

In order to obtain a better view of the extended specificities of these two enzymes a panel of recombinant protein substrates was used. The sequences were inserted in a linker region between two *E*.*coli* thioredoxin molecules by ligating double stranded oligonucleotides (encoding the actual target sequence) into a BamHI and a HindIII or SalI site of the vector construct (Figs 5A and 6A). For purification purposes a His_6_-tag was added to the C-terminal of the second thioredoxin (Fig 5A). A number of different potential substrate sequences were produced with this system, by ligating the corresponding oligonucleotides into the BamHI or HindIII/SalI sites of the vector. All of these substrates were expressed as soluble proteins in a bacterial host, *E*.*coli* Rosetta gami, and purified on IMAC columns to obtain a protein with a purity of 90–95%. Two different variants of the vector were used in this study, one having BamHI and HindIII sites for cloning of the linker region (as shown in Fig 5A) and one where the linker sites were BamHI and SalI. The SalI containing substrates (as shown in Figs 6 and 7) have an extra Asp residue in the linker region originating from the sequence of the SalI site. These recombinant proteins were then used to study the preference of the two enzymes (Figs 5, 6 and 7). Seven different constructs were produced with the first vector, containing the HindIII site, encoding the following sequences: the sequence identified as optimal for mouse GzmB (LIGFD↓VGVQ) [12], the sequence previously identified as the most optimal sequence for human GzmB (LIGAD↓VLVQ) [12], the consensus sequence obtained from an analysis of rat GzmB by phage display by Harris et al. (LIETD↓SGL) [13], a variant of the mouse GzmB consensus ‘variant 3’ (LIG**R**D↓VGVQ), mouse GzmB ‘variant 4’ (LI**ER**D↓VGVQ), the rMCP-2 consensus determined by phage display (VVLF↓SAVL) [12] and a control substrate with alternating Arg and Gly residues (GRGGRGGRG). The results from the analyses of these substrates showed both opossum and human GzmB preferred the rat GzmB consensus sequence. The rat GzmB consensus sequence contains two negatively charged amino acids in the P1 and P3 positions of the substrate. The cleavage of these substrates resulted in two clearly separated smaller bands, indicating cleavage only at one site in the middle of the linker sequence (Fig 5). In addition, both enzymes cleaved the mouse GzmB consensus almost equally well, whereas a marked difference in cleavage activity was seen for the human GzmB consensus (Fig 5). Human GzmB cleaved this substrate almost as efficiently as the rat GzmB consensus whereas opossum GzmB showed almost no cleavage of the same substrate (Fig 5E). A comparison of the activity of human GzmB against the rat and mouse GzmB consensus substrates showed the human enzyme cleaved the rat and the human consensus substrates approximately 10 times more efficiently that the mouse consensus substrate (Fig 5C). The opossum enzyme cleaved the rat consensus substrate 5–7 times better than the mouse with almost no cleavage of the human consensus substrate (Fig 5E). Using two substrates with no negatively charged residues in the linker region, including both the rMCP-2 consensus and a substrate with repeating Arg and Gly residues, no observable cleavage with either enzyme was seen. This provided a strong indication that these two enzymes were true asp-ases with clear specificities for negatively charged amino acids, which was also seen with the chromogenic substrates. Both enzymes were also very sensitive to positively charged amino acids close to the P1 Asp residue, indicating that both enzymes were relatively selective in their target specificity. Almost no detectable cleavage was seen with either enzymes with the mGzm variant 3 substrate where the single Asp is preceded by an Arg (Fig 5D and 5F). The same situation was seen for human GzmB with the mouse GzmB variant 4 substrate, where an Arg was inserted between the two negatively charged residues of this site (Fig 5D and 5F). The opossum showed some minor cleavage of this substrate, indicating that the two negatively charged residues partly compensated for the presence of the positively charged Arg residue (Fig 5F). In this analysis the opossum GzmB almost appeared more specific than the human variant. This finding partly contradicted the results from the chromogenic substrates where the opossum enzyme showed a relatively low selectivity between different asp-ase substrates (Fig 4).

To obtain more detail about the selectivity of these enzymes, the cleavage activity of these enzymes on a related set of substrates was analyzed. These altered substrates contained two negatively charged residues due to the presence of an additional Asp site in the cloning site of the 2x thioredoxin substrates previously described. This vector has been used in several previous articles investigating the cleavage specificities of a number of other mammalian serine proteases. The analysis of these substrates was more complicated as they contain additional negatively charged residues, however, they add additional important information to the understanding of the specificity of these enzymes. The mouse GzmB consensus substrate with the original SalI site (hence a second Asp in the sequence) was included in the first part of this analysis (first panels in Fig 5C and 5E). The human enzyme cleaved this substrate at almost the same rate as the new (HindIII) mouse consensus substrate (lacking a second Asp), whereas the opossum enzyme primarily cleaved at the second site leaving only one visible gel band due to the bands becoming equal in size. The cleavage of this substrate was estimated to be 3–5 better than the rat consensus substrate and almost 10 times better than the mouse consensus substrate lacking the second Asp residue (Fig 5E). Observing that opossum GzmB preferentially cleaved at the second site indicated that it could cleave more efficiently closer to the bulky thioredoxin molecule than the human enzyme, and was also possibly more affected by residues further away from the cleavage site.

### Use of an additional set of recombinant protein substrates containing an additional negative charged residue to obtain a more detailed picture of primary and extended specificities

As described above, in previous studies of protease cleavage specificity we have used a 2x thioredoxin vector with BamHI and SalI sites where the SalI site encodes an Asp residue (Fig 6A). A panel of such substrates was used to obtain additional information concerning the opossum and human GzmB (Figs 7 and 6 respectively). Human GzmB very efficiently cleaved the rat GzmB consensus sequence. By analyzing the protein band intensities of several gels with different enzyme concentrations we could estimate that human GzmB cleaved this sequence 3–5 times better than the sequence previously identified as the most optimal sequence for human GzmB. The sequence identified as optimal for mouse GzmB was favored even less. The cleavage of this substrate was estimated (using gel band densitometric measurements) to be 10–15 times less efficient than the rat GzmB consensus sequence (Fig 6). A small amino acid like alanine in the P2 position was preferred over a large aromatic amino acid such as phenylalanine, and as previously also shown; by inserting a basic amino acid in the P2 position between two negatively charged amino acids, the activity was dramatically reduced (Fig 6). Based on the absence of cleavage of the rMCP-2 consensus, the mMCP-5 consensus (VLLVSEVL) [14] and the human thrombin consensus (LTPRGVRL) [15, 16] we also concluded that this enzyme had a relatively narrow specificity (Fig 6). However, interestingly we found that the human enzyme switched to cleave at the second Asp site (within the SalI cloning site) when a basic amino acid was positioned next to the first Asp of the linker region (Fig 6D and 6E). However here, the cleavage rate was relatively low compared to the opossum GzmB. A somewhat unexpected result was the lack of cleavage of the rMCP-5 consensus sequence that contains two negatively charged residues: a Glu and an Asp (Fig 6F).

With these substrates the opossum GzmB showed a lower degree of specificity where we observed almost equally efficient cleavage of the human, mouse and rat consensus GzmB target sequences (Fig 7A). No cleavage was observed with the human thrombin substrate, containing a positively charged amino acid in the P1 position, as well as an Arg positioned close to the Asp residue of the SalI site (Fig 7D). However, a low level of cleavage was observed with both rMCP-2 consensus substrate and the mMCP-5 substrate (Fig 7D). These two sequences are chymase and elastase substrates, respectively. Interestingly, the rMCP-2 consensus sequence does not contain a negatively charged amino acid in the central part of the linker region. Instead opossum GzmB cleaved at the Asp residue of the SalI site (Figs 7D vs 5F). Human GzmB did not seem to cleave at this site, possibly due to a higher specificity and the location of this Asp residue close to the tightly folded second thioredoxin protein (Fig 6F). After cleavage at the second Asp by the opossum GzmB, both products were almost identical in size and therefore appeared as one single band on a SDS-PAGE gel (Fig 7). This was in contrast to the human enzyme, which only (or primarily) cleaved at the preferred centrally located sites thereby resulting in two differentially sized bands (Fig 6). This provided additional support reasoning that the opossum enzyme is slightly less specific than its human counterpart, although both had primary specificities for negatively charged amino acids (Fig 5). Two small bands for the rat GzmB substrate were also observed, which was probably due to cleavage at all three negatively charged amino acids within the linker region of this substrate, where the fragment containing the first thioredoxin becomes smaller and runs lower on the gel (Fig 7A). However, the surrounding of the Asp residue was still of importance as no cleavage of the thrombin substrate by the opossum GzmB was observed despite this substrate also containing the C terminally located Asp residue within the SalI site of the construct (Fig 7). All the reactions presented here were performed multiple times and often at different enzyme concentrations to validate the results and get comparative estimates. The 2x thioredoxin system has also recently been thoroughly validated in two articles, one on the cleavage specificity of human thrombin and the second on the exosite dependence of the same enzyme ([15, 16] See S1 Fig in the second (the exosite) article).

### Screening for the presence of potential GzmB specific protease inhibitors within the opossum genome

**Fig 8:**
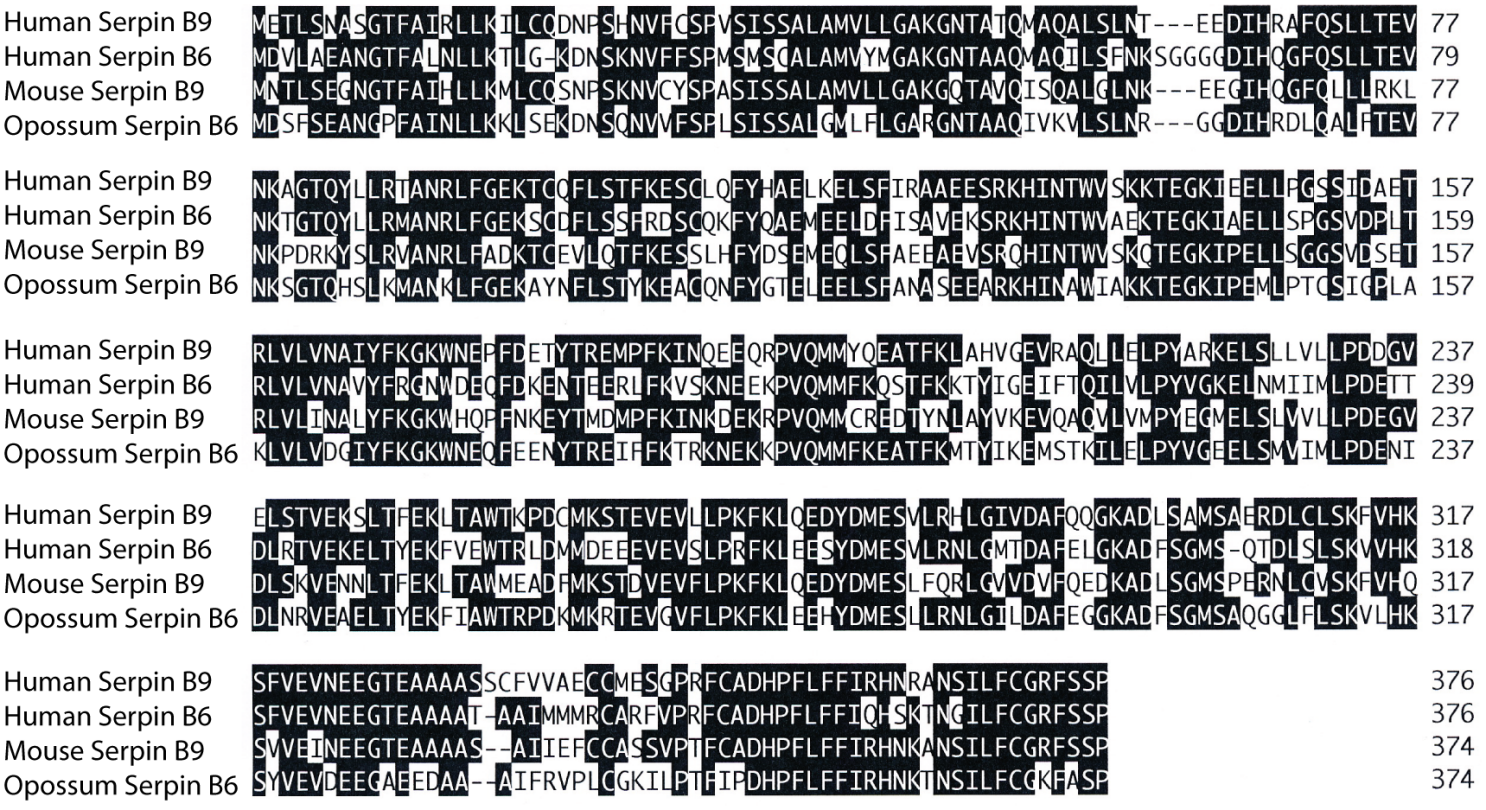
Alignment of human, mouse and opossum serpins. The amino acid sequences of the human serpin B9, human serpin B6, mouse serpin B9 and the closest homolog to these three sequences in the opossum, opossum serpin B6, were aligned using clustal W algorithm in the Lasergene program (DNASTAR Inc, USA). The opossum serpin B6 showed almost the same degree of sequence relatedness to human serpins B9 and B6 indicating that the opossum serpin B6 may have functions similar to both human serpins B6 and B9.

In placental mammals, GzmB activity is controlled by potent protease inhibitors belonging to the serpin family. The primary inhibitor of human GzmB is serpin B9 [17, 18]. To study the potential role of serpins in marsupials the opossum genome was screened for genes homologous to human and mouse serpin B9. The closest homolog identified was a gene named serpin B6. Alignment of human serpins B9 and B6, mouse serpin B9 and opossum serpin B6 showed that all had a very high degree of homology, where the opossum serpin B6 showed almost the same degree of sequence identity to both human serpin B6 and B9 (Fig 8). This suggests that the opossum has a serpin that may serve as a potent controller of the asp-ase activity hence affect the opossum enzyme. This indicates additional similarities between placental mammals and marsupials concerning these enzymes. However, for a more definite answer to this question the opossum serpin needs to be produced and tested for activity against the opossum GzmB enzyme.

## Discussion

The role of GzmB in the induction of caspase/Bid-dependent apoptosis in placental mammals is probably the most well established function of any of the hematopoietic serine proteases [4]. From an evolutionary perspective it is of interest to determine when this specificity appeared during vertebrate evolution. We can now show that an enzyme with a primary specificity for negatively charged amino acids was present before the separation of marsupials and placental mammals, at least 180 million years ago. This indicates that the function is old, with the question remaining, how old? To trace the enzymes further back in vertebrate evolution we have recently initiated a bioinformatic analysis of related genes in early mammals and non-mammalian vertebrates. It is well established that GzmB in all species examined is expressed from only one gene, and this gene is positioned at one end of the chymase locus [5–7]. The only non-placental mammal that this locus has been mapped in detail is the American opossum and there the locus contains two serine protease genes as previously described: a mast cell chymase and a second protease, which in phylogenetic analyses maps in the cluster of cathepsin G, granzymes, the duodenases and the mMCP-8 family of hematopoietic serine proteases [7]. However, based only on phylogenetics, it was difficult to assign it to any of these four subfamilies. By analyzing the cleavage specificity, we can now conclude that it is an asp-ase and therefore most likely a GzmB homolog. The characterization of the opossum enzyme as an asp-ase also provides strong indications that both the chymase and GzmB were the first two serine protease genes to appear in the chymase locus. Interestingly, in the context of the appearance of the asp-ase specificity, three genes with homology to chymase locus genes have been identified in the platypus. The platypus represents an earlier branch of mammalian evolution, the monotremes. This branch of egg-laying mammals harbors only 3 extant species: two species of anteaters; the echidnas, and the platypus. The three genes in the platypus genome that cluster with the chymase locus genes separate into two branches of the tree (Fig 1). Two of the genes, like the opossum chymase, cluster with the mast cell chymases (duodenase-like and GzmB, Fig 1) and the third with cathepsin G, granzymes, duodenases and mMCP-8 (GzmBCD like in Figs 1 and 2) indicating that this enzyme may also be a GzmB homolog. However, the three genes are found on two small contigs with no bordering genes, which makes it difficult to currently define them as part of a traditional chymase locus. Data from a larger bioinformatic analysis that we recently published indicates that the chymase locus may also exist in some reptiles, as exemplified by the Chinese alligator [19]. A gene distantly related to the mammalian chymase locus genes is also found in the clawed frog, *Xenopus tropicalis*, indicating that the locus may have appeared with early tetrapods, around 400 million years ago [19]. Recently, an independent analysis addressing the same questions concerning the origin of the hematopoietic serine proteases during vertebrate evolution has been published. This study also identifies the same platypus and *Xenopus* genes as described above [20].

We can conclude that an enzyme with asp-ase specificity has been part of mammalian immunity at least from early mammalian evolution and the branch point between marsupials, placental mammals and monotremes. The next step will be to try to specify the cleavage specificity of the three platypus enzymes, which can give us information of the situation 220–250 million years ago during an earlier stage of mammalian evolution. The analysis of the enzymes from the Chinese alligator and the clawed frog can also shed light on the early steps in the evolution of caspase/ Bid-dependent apoptosis mechanisms. Apoptosis is a key event in the defense against intracellular parasites, viruses and intracellular bacteria, but also of transformed tumor cells. An interesting question here is whether caspase/Bid-dependent and Bid-independent pathways have been accompanying the development of adaptive immunity from early jawed vertebrates, and in that case by which enzymes. Is this part of a divergent or convergent evolution, and have both pathways been part of our defense against intracellular parasites? Granzymes A and K are encoded from a separate locus, the T cell tryptase locus, and that locus seems to be the first of the four loci encoding hematopoietic serine proteases to appear [1, 19, 21]. These enzymes are already found in cartilaginous fish [19–21]. Granzyme A was long thought to primarily be involved in caspase-independent apoptosis induction. However, recently this notion was challenged when more physiological concentrations of the enzyme were used to study its function [22, 23]. At these levels it induced cytokine production but not apoptosis, therefore the question remains regarding the primary role of this enzyme and its role in caspase-independent apoptosis. However, what is intriguing is that these two enzymes have been conserved during more than 450 million years of vertebrate evolution, which indicates a very important function in immunity [20, 21]. The question is now when during vertebrate evolution did caspase/Bid-dependent mechanisms, governed by asp-ase specific serine proteases, become an important immune mechanism in our defense against tumor cells and cells infected with intracellular parasites. An alternative scenario is that enzymes with other primary and extended specificities are performing the same function as GzmB is in mammals, and that similar mechanisms have developed in earlier vertebrates by convergent evolution. These questions will need to be addressed.

The collected information from the chromogenic and recombinant substrate analyses resulted in a relatively complex picture of the substrate specificity of the human and opossum GzmBs. From the chromogenic assays the opossum GzmB seems to have a broader specificity as it relatively efficiently cleaves all three Asp substrates whereas the human enzyme only cleaves one efficiently, the IEPD substrate, and a second the VEID with much lower efficiency (Fig 4). A quite different picture then emerges from the analysis of the first set of recombinant substrates, with a HindIII site. There opossum GzmB appeared more specific than the human enzyme as it only cleaved the mouse and rat consensus substrates and not the human consensus substrate (Fig 5). When the substrates containing an extra Asp residue originating from the SalI site were analyzed, the opossum enzyme again appeared more broad. It frequently cleaved close to the thioredoxin domains and it also relatively efficiently; almost all of the different substrates except the thrombin, where a positively charged Arg residue is positioned close to the Asp of the SalI site (Fig 7D). This provides important information for the analysis of extended specificity studies. Residues relatively far from the actual cleavage site can be of major importance for the efficiency of cleavage. The number of potential sites in the region as well as how open the region is for protease access are also of major importance for efficient cleavage by both enzymes. Based on these findings the opossum enzyme seems to have a slightly broader specificity compared to its human counterpart and is also able to cleave more efficiently close to tightly packed domains like the thioredoxin domains than the human enzyme. A study on the evolution of the cleavage specificities of the different subfamilies within the trypsin/chymotrypsin family of serine proteases can put the differences in extended specificity of these two enzymes in an evolutionary context [24]. This study has shown that changes in primary specificities have occurred primarily in two subfamilies: the pancreatic chymotrypsin-elastase subfamily and the hematopoietic serine proteases [24]. This latter subfamily is also named the immune defense proteases (IDP) or the granule associated proteases of immune defense (GASPIDS) to which both of the enzymes of this study belong [20, 24]. Interestingly, the subfamily with the highest variability of these two is the latter subfamily, the immune proteases. In mammals, the members of this subfamily that are encoded from the chymase locus have a specific characteristic as they have lost a cysteine bridge that is positioned close to the active site of all other members of this protease family. These proteases show a remarkable plasticity concerning both primary and extended cleavage specificities. Mutations within descendants from the first ancestral enzyme of the chymase locus have resulted in the appearance of all major primary specificities, i.e., chymases, asp-ases, elastases and tryptases. This diversification may have started already 350–400 million years ago during early tetrapod evolution. However, the most dramatic expansion in the number of genes within the locus and the variation in specificities has occurred during late mammalian evolution [1, 5, 7, 20]. The loss of a cysteine bridge may have had a major impact on this process by increasing the flexibility in the region of active site, facilitating the generation of new specificities and also changes in activity. It has been calculated that this loss of a cysteine bridge broadens the neck of the active site pocket by approximately 1Ångström [24].

Studies of three amino acid positions in the enzyme; residues 189, 216 and 226, according to chymotrypsinogen numbering, which are located in the active site pocket of the enzyme have also been shown to have predictive value on the primary specificity of these enzymes. Almost all tryptases have a triplet of DGG, where the Asp favors the basic amino acids Arg and Lys in the P1 position, most likely due to direct electrostatic interaction [24]. In general, the chymases have a triplet of SGA with a few exceptions: mMCP-1 a TGA, rMCP-2 an AGA and rMCP-4 LGI. Although differing slightly in sequence of this triplet, all three of the latter proteases are true chymases and they all display a highly specific extended specificity [25–27]. Human, rat and mouse GzmB, the asp-ases, have TGR, AGR and AGR, respectively (Fig 2). Interestingly, changing the Arg in the triplet of rat GzmB to an Glu changes the primary specificity of the enzyme to a tryptase [28]. Mutating Arg192 in rat GzmB to an E reversed the P3 specificity of this enzyme to Arg instead of Glu showing the importance of the amino acids in and around the pocket for both primary and extended specificities [13]. Recently we have also shown the importance of both Arg143 and Lys192 for the extended specificity, and specifically the preference for negatively charged amino acids in the P2´position of the human mast cell chymase [29]. In line with these findings, exchanging the triplet of a theoretically ancestral chymase from SGA to DGG was expected to turn the enzyme into a tryptase [24]. However, this enzyme still remains primarily a chymase with a slightly increased activity towards tryptase substrates [24]. Here, the triplet can give some guidance to the primary specificity but other residues are clearly of importance. The opossum GzmB has a triplet that is identical to mouse and rat GzmB: AGR (Fig 2). However, what causes the difference observed for the extended specificity of the opossum GzmB is difficult to tell from only the primary sequence. A detailed structural analysis, possibly involving a set of mutants, would be needed to address this question. The difference in extended specificity between the human and opossum enzymes while maintaining the same primary specificity is interesting from a functional perspective. The apparent flexibility in both extended and primary specificities within this subfamily shows the power of the evolutionary adaptation within this protease subfamily. However, what these changes in primary and/or extended specificities and activities have on the general functions of these enzymes and specifically for apoptosis induction by caspase or Bid cleavage by the GzmB homologs are poorly known and need to be addressed further.

## Supporting Information

S1 Fig A sample of the recombinant opossum GzmB preparation was cleaved with trypsin and separated by mass spectroscopy. Several of the internal peptides were identified, confirming the protein identity as opossum gzmB.
(TIF)

S2 Fig Cleavage analyses of five additional chromogenic substrates (two tryptase, one leucine, one chymase and one elastase) by opossum GzmB. The very low activity seen with all three enzymes against the tryptase substrates is due to low levels of remaining enterokinase used in the activation of the recombinant enzymes.
(PDF)
