# Supplementary figures and images for: Asp-ase Activity of the Opossum Granzyme B Supports the Role of Granzyme B as Part of Anti-Viral Immunity Already during Early Mammalian Evolution
Source: PLoS One. 2016 May 6;11(5):e0154886. doi: 10.1371/journal.pone.0154886 (PMC4859502; doi:10.1371/journal.pone.0154886)

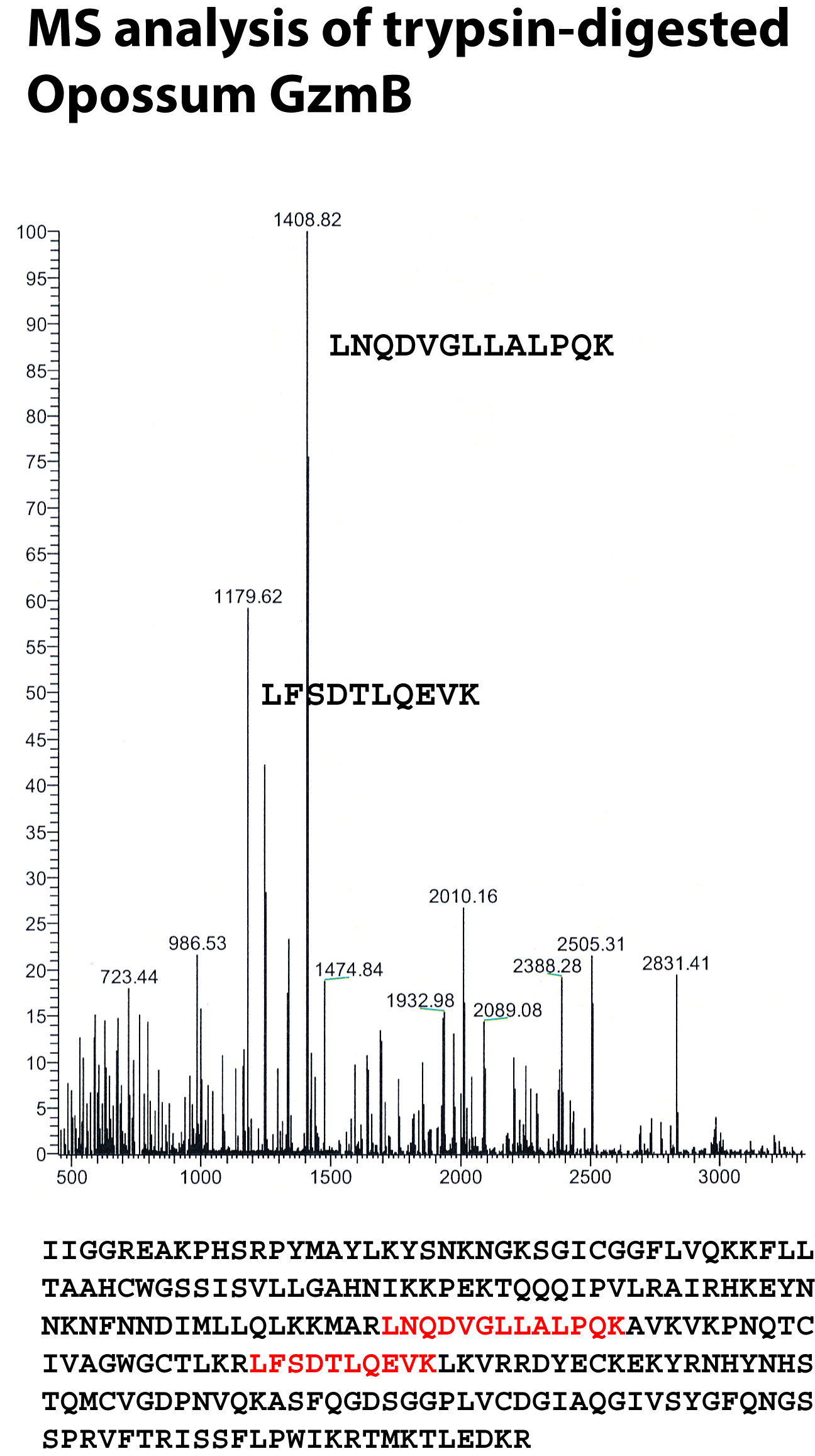

Supplement: S1 Fig — Several of the internal peptides were identified, confirming the protein identity as opossum gzmB. (TIF) [file pone.0154886.s001.tif]

Absorbance at 405nm

**A**

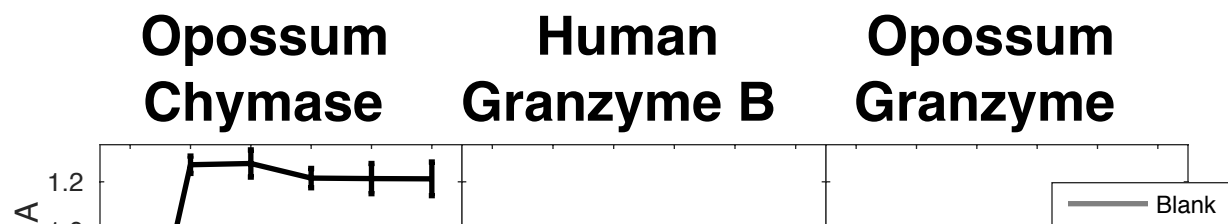

**B**

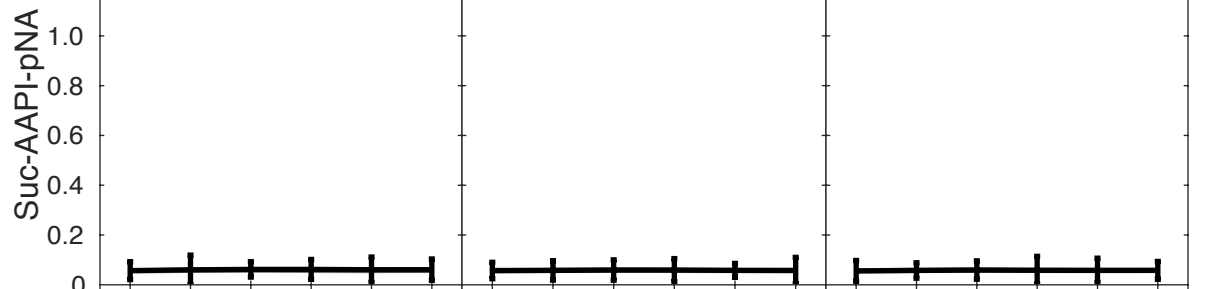

**C**

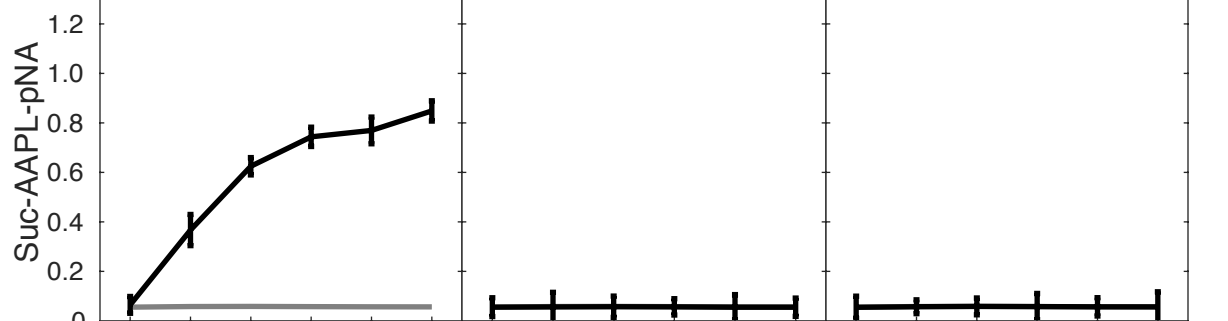

**D**

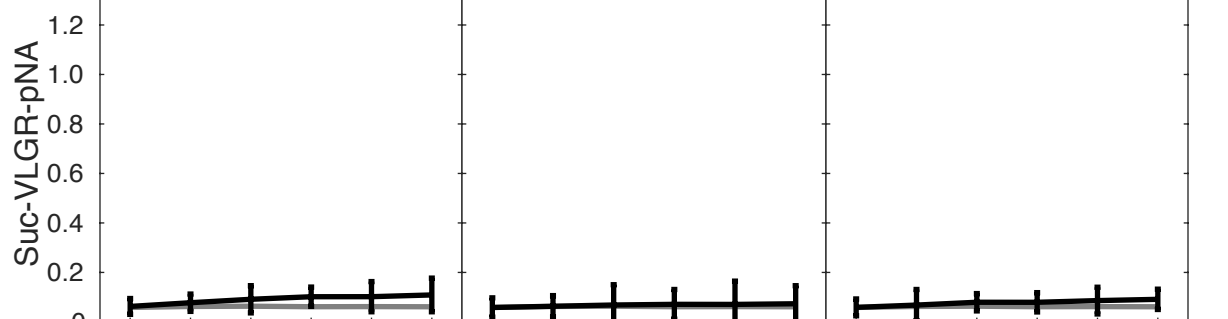

**E**

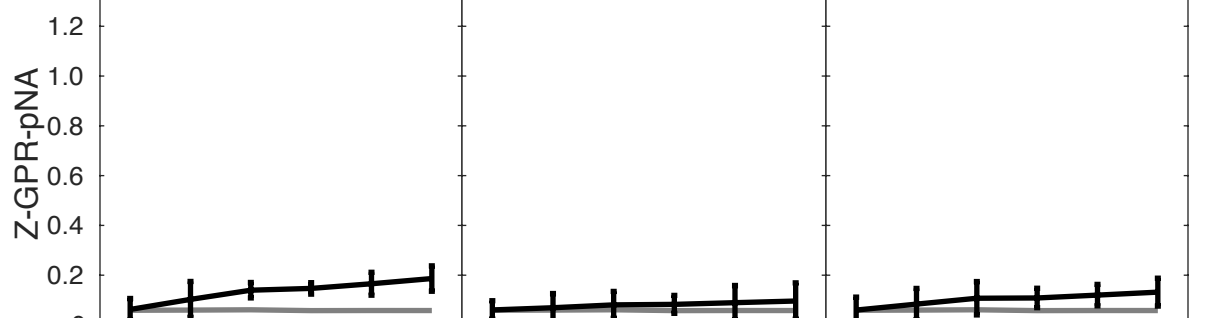

Time(h)

Supplement: S2 Fig — The very low activity seen with all three enzymes against the tryptase substrates is due to low levels of remaining enterokinase used in the activation of the recombinant enzymes. (PDF) [file pone.0154886.s002.pdf]
